# Competitive Androgen Receptor Antagonism as a Factor Determining the Predictability of Cumulative Antiandrogenic Effects of Widely Used Pesticides

**DOI:** 10.1289/ehp.1205391

**Published:** 2012-09-10

**Authors:** Frances Orton, Erika Rosivatz, Martin Scholze, Andreas Kortenkamp

**Affiliations:** 1 Centre for Toxicology, School of Pharmacy, London, United Kingdom; 2 Institute of Chemical Biology, Imperial College London, London, United Kingdom

**Keywords:** antiandrogen, AR-antagonism, concentration addition, endocrine disruption, fungicide, mixture, pesticide

## Abstract

Background: Many pesticides in current use have recently been revealed as *in vitro* androgen receptor (AR) antagonists, but information about their combined effects is lacking.

Objective: We investigated the combined effects and the competitive AR antagonism of pesticide mixtures.

Methods: We used the MDA-kb2 assay to test a combination of eight AR antagonists that did not also possess AR agonist properties (“pure” antagonists; 8 mix: fludioxonil, fenhexamid, *ortho*-phenylphenol, imazalil, tebuconazole, dimethomorph, methiocarb, pirimiphos-methyl), a combination of five AR antagonists that also showed agonist activity (5 mix: cyprodinil, pyrimethanil, vinclozolin, chlorpropham, linuron), and all pesticides combined (13 mix). We used concentration addition (CA) and independent action (IA) to formulate additivity expectations, and Schild plot analyses to investigate competitive AR antagonism.

Results: A good agreement between the effects of the mixture of eight “pure” AR antagonists and the responses predicted by CA was observed. Schild plot analysis revealed that the 8 mix acted by competitive AR antagonism. However, the observed responses of the 5 mix and the 13 mix fell within the “prediction window” boundaries defined by the predicted regression curves of CA and IA. Schild plot analysis with these mixtures yielded anomalous responses incompatible with competitive receptor antagonism.

Conclusions: A mixture of widely used pesticides can, in a predictable manner, produce combined AR antagonist effects that exceed the responses elicited by the most potent component alone. Inasmuch as large populations are regularly exposed to mixtures of antiandrogenic pesticides, our results underline the need for considering combination effects for these substances in regulatory practice.

Certain pesticides are known to disrupt male sexual differentiation *in vivo* by antagonizing the androgen receptor (AR) (Gray et al. 1994; Lambright et al. 2000; Ostby et al. 1999) or by interfering with steroid-converting enzymes in fetal life (Blystone et al. 2007; Vinggaard et al. 2005). These pesticides can act together to produce combination effects (Christiansen et al. 2008; Vinggaard et al. 2005), which can also occur when combined with other chemicals known to disrupt androgen action (Rider et al. 2008, 2009). Data from food residues indicate that there is a potential for simultaneous human exposure to at least some of these pesticides.

We previously reported that a number of current-use pesticides are antiandrogenic (Orton et al. 2011). Using these data, we formulated mixtures based on the most common pesticides present in foods in Europe. Many of these pesticides are also commonly found in the United States (e.g., fludioxonil, in 26% of strawberries and 14% of grapes; fenhexamid, in 24% of strawberries; ortho-phenylphenol, in 34% of oranges; dimethomorph, in 28% of lettuces; cyprodinil, in 27% of grapes; pyrimethanil, in 31% of strawberries; chlorpropham, in 76% of potatoes) (U.S. Environmental Protection Agency 2011). Considering that risk assessment procedures do not currently account for mixture effects, it is possible that risks to male reproductive health by pesticides are being underestimated. Although antiandrogenic mixture effects have been described for certain pesticides, some of which are obsolete (Birkhoj et al. 2004; Kjærstad et al. 2010; Nellemann et al. 2003), similar data with more widely used pesticides are lacking. Because many current-use pesticides act as AR antagonists *in vitro* (Kojima et al. 2004; Orton et al. 2009, 2011), it is plausible to assume that these pesticides might also have mixture effects. However, empirical evidence to support this idea is lacking. Because none of the pesticides chosen for our mixture studies were tested *in vivo*, it was important to investigate whether these substances have the ability to act jointly at the receptor level *in vitro*. If that was found to be the case, it would create alerts for prioritization of *in vivo* testing.

This is all the more relevant because of indications of negative effects on male reproductive health from epidemiological studies of occupational pesticide exposures. For example, statistically significant associations between genital malformations or decreased penile length in boys with occupational maternal or paternal pesticide exposure have been observed in the Netherlands (Pierik et al. 2004), Denmark (Andersen et al. 2008; Wohlfahrt-Veje et al. 2012), and France (Gaspari et al. 2011) and also in a meta-analysis of hypospadias incidence in several countries (Rocheleau et al. 2009). However, these studies could not identify specific pesticides as being involved in the analyzed effects.

At present, there are 1,252 registered active ingredients in pesticide formulations in the United States (U.S. EPA, personal communication). There are 411 registered entities in Europe, with another 72 pending registration (European Commission 2011). With such a high number of registered active substances, it is practically impossible to test all possible combinations to arrive at robust conclusions about the nature of combination effects. Therefore, exploring the accurate predictability of mixture responses using modeling approaches is essential. Mixture modeling uses single compound testing data to describe the effects of simultaneous exposures to multiple chemicals, with the aim of replacing or significantly reducing testing for the prohibitively large number of chemicals and combinations present in the environment. In this context, modeling approaches work under the hypothesis that compounds elicit their effects without affecting the toxicity of other mixture components, i.e., the additivity assumption (reviewed by Kortenkamp 2007). Two concepts are commonly used to explore the additivity assumption: *a*) concentration addition (CA, also called dose addition), and *b*) independent action (IA, also called response addition). For CA, it is assumed that all compounds have a similar mechanism of action (e.g., binding the same receptor), whereas for IA, it is assumed that all mixture components affect the same end point via different sites or modes of action (i.e., using a dissimilar mechanism of action). Both additivity models assume no interaction between the compounds, neither on a physicochemical level nor in their toxicokinetics and toxicodynamics.

CA has consistently been shown to be a good model for predicting antiandrogenic effects [e.g., *in vivo* (Christiansen et al. 2008; Hass et al. 2007; Howdeshell et al. 2008) and *in vitro* (Ermler et al. 2011)]. To our knowledge, there are only two examples where CA has failed to predict the mixture effect. A significant deviation (synergism) was observed in response to five antiandrogenic parabens *in vitro* (Kjaerstad et al. 2010) and to four antiandrogenic contaminants *in vivo* [di(2-ethylhexyl) phthalate; two fungicides present in food, vinclozolin and prochloraz; and a pharmaceutical, finasteride] (Christiansen et al. 2009). To investigate the predictability of mixtures of AR antagonists using the MDA-kb2 cell assay, and considering the features of this assay, we hypothesized that CA and not IA would be the appropriate prediction concept (for an overview see Ermler et al. 2011).

Some AR antagonists can stimulate the receptor, sometimes at concentrations higher than those required for antagonism and, in other cases, over the same concentration range (Ermler et al. 2011; Orton et al. 2011). Many AR antagonists are not capable of eliciting AR agonist effects, and these are referred to as “pure” antagonists. The antagonist/agonist activity of some antiandrogens is thought to be due to different actions on the AR receptor whereby the AR is simultaneously stimulated by binding to a distinct domain of the receptor (Tamura et al. 2006). However, it is not known how such effects might affect the predictability of mixture models and whether the “similarity” criterion of CA is fulfilled under these circumstances. Therefore, we investigated whether CA was a suitable prediction tool for mixtures regardless of mixture composition, or whether mixtures composed of antagonist/agonist antiandrogens produced responses that deviated from CA. We used a Schild plot analysis to distinguish the similarity requirements for both scenarios. This pharmacological method allowed us to assess whether the observed antiandrogenic activity was solely due to competitive antagonism of dihydrotestosterone (DHT) binding to the ligand binding domain of the AR (Kenakin 1993).

## Methods

*Test compound selection.* We have previously shown that 24 current-use, environmentally relevant pesticides were AR antagonists (Orton et al. 2011), and our mixture selection for the present study was based on these data. For the 24 that were antiandrogenic, we ranked the pesticides by their environmental relevance ratio (ERR), a measure of combined potency and prevalence and excluded those with lapsed registration status (as of January 2010) and cytotoxicity at ≤ 10 µM. Twelve pesticides fulfilled these criteria, in order of ERR: dimethomorph (ERR 45.6; re-registration date September 2017), fludioxonil (31.2; October 2018), fenhexamid (11.9; December 2015), imazalil (9.9; December 2021), linuron (6.9; December 2013), *ortho*-phenylphenol (6.1; December 2019), pirimiphos-methyl (5.5; September 2017), tebuconazole (5.5; August 2019), chlorpropham (2.9; June 2015), methiocarb (2.5; September 2017), cyprodinil (2.2; April 2017), and pyrimethanil (1.0; May 2017). In addition, vinclozolin was included because of its high ERR (79.8), its known *in vivo* potency (e.g. Gray et al. 1994), and its continued detection in foodstuffs in Europe {0.38% in 2008 [(European Food Safety Authority (EFSA) 2010] and 0.2% in 2009 (EFSA 2011)}, despite its expired registration status of January 2007.

*Chemicals.* DHT (> 97% purity) was purchased from Steraloids Ltd. (Croydon, Surrey, UK); dimethomorph and methiocarb (> 98.7% purity were purchased from Greyhound Chromatography and Allied Chemicals; Birkenhead, Merseyside, UK); and all other pesticides (> 97% purity) were purchased from Sigma-Aldrich (Poole, Dorset, UK). All test compounds were dissolved in > 99.7%-purity ethanol to make stock solutions to be used in the assays.

*MDA-kb2 assay.* MDA-kb2 cells [catalog number CRL-2713; American Tissue Culture Collection (ATCC), Manassas, VA, USA] are human breast cancer cells stably transfected with a firefly luciferase reporter gene that is driven by an androgen–response element-containing promoter (Wilson et al. 2002). Details of the modified assay were published previously (Ermler et al. 2010). Briefly, cells were seeded at a concentration of 1 × 10^5^ cells/mL in phenol red–free Leibowitz-15 medium (Invitrogen Ltd., Paisley, UK) containing 10% charcoal-stripped fetal calf serum (Invitrogen Ltd.) in white luminometer plates. After 28 hr, luciferase activity was determined with SteadyGlo assay reagent (Promega UK Ltd., Southampton, Hampshire, UK) and measured in a plate reader (FLUOstar Optima; BMG Labtech GmbH, Offenburg, Germany). For regression analysis, cells were exposed to eight serial dilutions of selected pesticides with or without DHT (0.25 nM). Subsequent to the initial testing range of 1.17 nM–150 µM, the mixtures’ concentrations were modified to reflect the potency and toxicity of each individual mixture. For Schild plot analysis, cells were coexposed with eight serial dilutions of DHT (0.009–20 nM) and fixed concentrations of pesticide mixtures (150–6.25 µM), which varied according to the individual activity/toxicity of each mixture. For all testing scenarios, the following controls were run on each plate: medium, ethanol (0.25%), DHT coexposure (0.25 nM), DHT serial dilutions (0.009–20 nM) with ethanol (0.25%), and procymidone (0.005–3.2 µM) with DHT (0.25 nM). All concentrations were tested in duplicate over two plates; each mixture stock was measured at least twice in separate experiments, and mixtures were independently tested at least three times (using new stock solutions, in separate experiments) by two experimenters. For comparative purposes, luminescence was normalized to DHT alone at the coexposure concentration (i.e., maximum response, 100%) and solvent-only (ethanol) controls (i.e., minimum response, 0%).

*Cytotoxicity as a confounding factor.* The MDA-kb2 assay measures decreases in luminescence of the DHT agonist that occur as a result of receptor antagonism. Because the luminescence signal can also be driven down by cytotoxicity, it is important to distinguish antagonism from interfering cytotoxicity, and we adopted well-established procedures (Ermler et al. 2010, 2011; Korner et al. 2004) to deal with this issue. Briefly, cytotoxicity was determined in treatments without DHT by a reduction in luminescence relative to the ethanol controls. Where agonism in the absence of DHT was observed, the comparison was with the maximal response.

Renilla *assay.* We constructed a *Renilla* luciferase plasmid with a mammalian selection marker and a constitutively active promoter [the herpes simplex virus–thymidine kinase (*HSV-Tk*) gene] in order to eliminate the possible interfering effects of cell proliferation. Briefly, 4 µg DNA was incubated with 6 µL TurboFect (Fermentas Gmbh, St. Leon-Rot, Germany) in 400 µL of serum-free Leibowitz L-15 medium (Invitrogen Ltd.) for 20 min. MDA-kb2 cells were transfected with the *Renilla* construct for 48 hr prior to following the normal procedure for the MDA-kb2 assay. In order to read both the luciferase and *Renilla* signals, after 28 hr of incubation, luciferase activity was determined with Dual-Glo Reporter assay reagent (Promega UK Ltd.), which employs the sequential addition of two reconstituted reagents with luminescence measurement after each reagent addition (FLUOstar Optima, BMG Labtech GmbH). The first reagent provides the necessary substrate for firefly luciferase, and the second reagent quenches this activity while at the same time activating *Renilla* luciferase. Cells transfected with the *Renilla* construct were exposed to the 5 mix IC_10_ only; for regression analysis, 5 mix (serial dilutions: 150–5.6 µM) was coexposed with a fixed concentration of DHT (0.25 nM), and for Schild plot analysis, serial concentrations of DHT (0.009–20 nM) with various fixed concentrations of 5 mix (110–13.75 µM).

*Test mixtures.* All mixtures were designed as fixed-ratio equipotent mixtures. We tested three distinct pesticide mixtures: an 8 mix, a 5 mix, and a 13 mix. The 8 mix comprised eight “pure” AR antagonists (fludioxonil, fenhexamid, *ortho*-phenylphenol, tebuconazole, dimethomorph, imazalil, methiocarb, pirimiphos-methyl); the 5 mix comprised five antagonists with additional agonist properties (cyprodinil, pyrimethanil, vinclozolin, chlorpropham, linuron); and the 13 mix comprised the eight “pure” antagonists together with the five “mixed” antagonists. Fixed-mixture ratios were calculated in proportion to the concentrations of the individual mixture components that led to a suppression of DHT effects by 1%, 10%, 20%, or 50% [here termed inhibitory concentrations (ICs) IC_01_, IC_10_, IC_20_, IC_50_]. The 13 mix was tested at four fixed mixture ratios (IC_01_, IC_10_, IC_20_, IC_50_), and the 8 mix and 5 mix were tested at two fixed mixture ratios (IC_01_, IC_10_) [see Supplemental Material, Table S1 (http://dx.doi.org/10.1289/ehp.1205391)]. The mathematical and statistical procedures used for calculating mixture effects according to CA and IA are described by Faust et al. (2001).

*Schild plot calculations.* To confirm applicability of the MDA-kb2 assay to Schild plot analysis, we first determined concentration–effect curves for the agonist DHT in the presence of various concentrations of flutamide. From the concentration–effect curves, we estimated a series of concentration ratios (i.e., the ratio of the DHT concentration to produce a specific effect in the presence of the antagonist to the concentration required in the absence of the antagonist) for a given effect. This was determined for several concentrations of the antagonists. To get the most accurate results, we used a 50% inhibition—the concentration ratio calculated as the IC_50_ in the presence of antagonist divided by the IC_50_ in the absence of antagonist. The Schild plot analysis was then based on the linear regression:

log(IC_50_^^DHT + A^^/IC_50_^^DHT^^ – 1) = –log(*K_D_*) + θ × log(*c_A_*). [1]

Here, *K_D_* is the (unknown) dissociation constant of the antagonist, *c_A_* the concentration of the antagonist “A” held fixed in the experiments, and θ the slope parameter. The unknown parameters, θ and log(*K_D_*), were estimated by ordinary least squares. If the regression is linear with a slope of 1, the antagonism is competitive and, by definition, the agonist and antagonist act at the same sites (Kenakin 1993). The concentration–response curves recorded in the presence of a fixed concentration of the antagonist will shift to the right of the DHT curve, with the same maximum response and (generally) the same shape. Therefore, we always used the logit model for the data analysis and performed Schild regression analysis only when the assumption of similar maximum responses was justified. The same principles were applied to the pesticide mixtures.

*Statistics.* To analyze AR antagonist action, raw luminescence readings were normalized on a plate-by-plate basis to the means of the positive DHT controls (*n* = 8) and the solvent controls (*n* = 8), which were placed on the same plate. Luminescence readings from pesticides tested in the absence of DHT were divided by the mean of the solvent controls from the same plate and analyzed for negative and positive trends (suggestive of cytotoxic or androgenic action, respectively). All data from the same test compound were pooled and statistical concentration–response regression analyses were conducted by using the best-fit approach to derive ICs for androgenicity (Scholze et al. 2001). To control for variations between experiments, concentration–response data were analyzed by using a generalized nonlinear mixed modeling approach (Vonesh and Chinchilli 1996) with plate as a random effect modifier for individual effect data. If readings in the absence of DHT showed indications for cytotoxic or androgenic action, the nonmonotonic concentration–response relationship was modeled by nonparametric local regression methods (Cleveland et al. 1988). From this robust fitting method, we derived effect concentrations (ECs) for androgenicity, with a 10% increase over the mean solvent mean as the minimum effect criterion, and ECs for cytotoxicity (if present) as 10% reduction of the maximal observed androgenic action. Data points associated with cytotoxicity were not included in regression analysis for antiandrogenicity. Differences between predicted and observed effect doses were deemed statistically significant when the 95% confidence intervals (CIs) of the prediction did not overlap with those of the experimentally observed mixture effects. All statistical analyses were performed using SAS statistical software version 9.2 (SAS Institute Inc., Cary, NC, USA).

## Results

**Figure 1:**
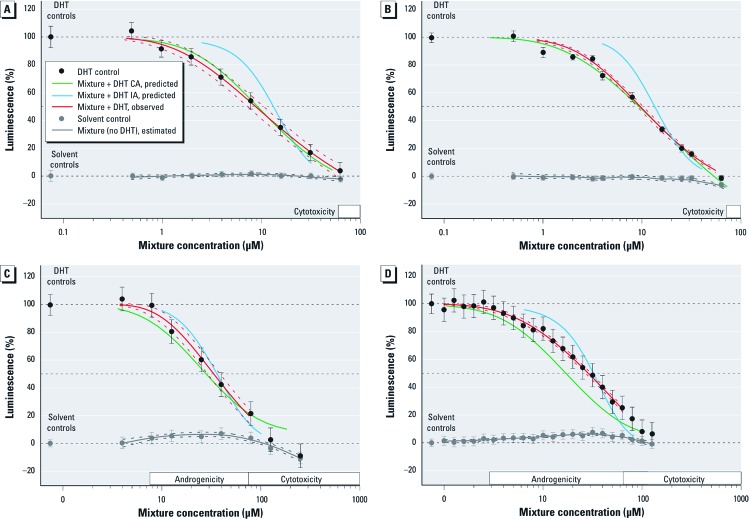
Predicted and observed antiandrogenic activity (mean responses ± SDs) of mixtures with 8 pesticides composed in the ratio of their individual IC_10_ values (*A*) and IC_01_ values (*B*), with 5 pesticides mixed in the ratio of their individual IC_01_ values (*C*), and with 13 pesticides mixed in the ratio of their individual IC_10_ values (*D*). Observed mixture effects are from at least three independent mixture experiments and shown as mean ± SD, predicted effect curves were calculated using the model of CA and IA. Regression fit of the observed effects is shown as solid red line, with the dotted red lines indicating the corresponding 95% CI. Estimated mean effect (solid gray line) and 95% CI (dotted gray lines).

Low variation between experiments, good repeatability, and complete regression curves for all the selected individual pesticides meant that all compounds were suitable for mixture assessment. All mixtures showed AR antagonist activity in a clear dose-dependent way (Figure 1). The agreement between observed and predicted AR antagonistic activity for a given mixture is shown for two selected response levels in Table 1: IC_50_s for the mixtures were only once overestimated by both CA and IA (13 mix, IC_01_ mixture ratio, 10% inhibition) and in all other cases were never outside the range predicted by CA and IA. Cytotoxicity was only observed at high mixture concentrations (8 mix: EC_10_, 60–77 µM; 5 mix: EC_10_, 70–74 µM; 13 mix; EC_10_, 63–81 µM) [for more information, see Supplemental Material, Table S2 (http://dx.doi.org/10.1289/ehp.1205391)]. The overlap with antiandrogenic responses was negligible and did not interfere with the detection of AR antagonistic responses (Figure 1A–D). The model parameters, together with estimated AR antagonist concentrations and effect concentrations for androgenicity and cytotoxicity are listed in Supplemental Material, Table S2. Cytotoxicity data for all test mixtures are also shown in Supplemental Material, Figure S2.

**Table 1:** Statistical uncertainty of predicted and observed ICs [means (95% CIs)] for mixtures.

Inhibition level *x*	Inhibition concentration IC_X_(mixture) [M]										
	Observed			Predicted by CA			Predicted by IA		
8 pesticides (IC01)a												
10%	1.65E-6	(1.11E-6, 2.21E-6)	2.12E-6	(1.87E-6, 2.29E-6)	5.47E-6	(4.10E-6, 6.18E-6)*
50%	9.74E-6	(7.91E-6, 1.07E-5)	9.66E-6	(9.00E-6, 1.03E-5)	1.35E-5	(1.20E-5, 1.46E-5)*
8 pesticides (IC10)a												
10%	1.49E-6	(1.23E-6, 1.85E-6)	1.87E-6	(1.64E-6, 2.03E-6)	4.57E-6	(3.46E-6, 5.26E-6)*
50%	8.79E-6	(7.55E-6, 1.04E-5)	9.13E-6	(8.53E-6, 9.76E-6)	1.32E-5	(1.17E-5, 1.45E-5)*
5 pesticides (IC01)a												
10%	1.03E-5	(8.25E-6, 1.21E-5)	6.73E-6	(5.86E-6, 7.82E-6)*	1.39E-5	(1.13E-5, 1.66E-5)
50%	3.35E-5	(2.95E-5, 3.78E-5)	2.80E-5	(2.58E-5, 3.09E-5)	3.60E-5	(3.28E-5, 4.10E-5)
5 pesticides (IC10)a												
10%	8.97E-6	(6.77E-6, 1.20E-5)	6.38E-6	(5.47E-6, 7.28E-6)	1.33E-5	(9.93E-6, 1.67E-5)
50%	3.54E-5	(3.11E-5, 4.06E-5)	2.89E-5	(2.68E-5, 3.19E-5)	3.77E-5	(3.41E-5, 4.32E-5)
13 pesticides (IC01)a												
10%	5.56E-6	(4.38E-6, 7.39E-6)	3.89E-6	(3.55E-6, 4.16E-6)*	1.38E-5	(1.06E-5, 1.52E-5)*
50%	2.61E-5	(2.38E-5, 2.95E-5)	1.70E-5	(1.61E-5, 1.80E-5)*	3.11E-5	(2.83E-5, 3.37E-5)
13 pesticides (IC10)a												
10%	5.20E-6	(3.47E-6, 7.28E-6)	3.61E-6	(3.24E-6, 3.88E-6)	1.11E-5	(8.24E-6, 1.27E-5)*
50%	2.86E-5	(2.68E-5, 3.01E-5)	1.71E-5	(1.63E-5, 1.80E-5)*	3.14E-5	(2.81E-5, 2.81E-5)
13 pesticides (IC20)a												
10%	3.25E-6	(2.28E-6, 4.91E-6)	3.48E-6	(3.14E-6, 3.75E-6)	1.01E-5	(7.30E-6, 1.17E-5)*
50%	2.42E-5	(2.09E-5, 2.89E-5)	1.71E-5	(1.63E-5, 1.80E-5)*	3.06E-5	(2.72E-5, 3.35E-5)
13 pesticides (IC50)a												
10%	5.41E-6	(3.70E-6, 7.44E-6)	3.35E-6	(2.99E-6, 3.64E-6)*	9.14E-6	(6.47E-6, 1.08E-5)
50%	3.24E-5	(2.70E-5, 3.67E-5)	1.75E-5	(1.65E-5, 1.85E-5)*	2.89E-5	(2.57E-5, 3.20E-5)
aSee Supplemental Material, Table S1 (http://dx.doi.org/10.1289/ehp.1205391) for mixture ratios. *Statistically significant compared with observed ICs. Significance between predicted and observed ICX values was judged as a non-overlapping of their 95% percentile bootstrap CIs.												

There was good agreement of the 8 mix responses with those predicted by CA over the entire concentration range and for both tested mixture ratios (Figure 1A,B, Table 1). This mixture was composed entirely of “pure” AR antagonists. However, CA consistently overestimated the combined effects of mixtures containing AR antagonists that also showed AR agonistic properties (5 mix, Figure 1C; 13 mix, Figure 1D). With these two mixtures, we observed androgenic activity at low concentrations when tested in the absence of DHT [see Supplemental Material, Table S2 (http://dx.doi.org/10.1289/ehp.1205391)]. When tested on their own, none of the individual pesticides in the mixtures showed AR agonistic effects at their concentration in the mixture. The androgenicity of 5 mix and 13 mix therefore appears to be a genuine combination effect. Indications for toxicity were detected only at high tested concentrations and are unlikely to interfere with the mixture assessment.

By performing Schild plot analysis with the pure antiandrogen flutamide and DHT, we were able to confirm competitive receptor antagonism. Increasing concentrations of this antagonist shifted the dose–response curve of the agonist DHT to the left [see Supplemental Material, Figure S1A,B (http://dx.doi.org/10.1289/ehp.1205391)], indicating that agonist and antagonist acted in a competitive manner at the same receptor site (i.e., the ligand-binding domain of the AR).

**Figure 2:**
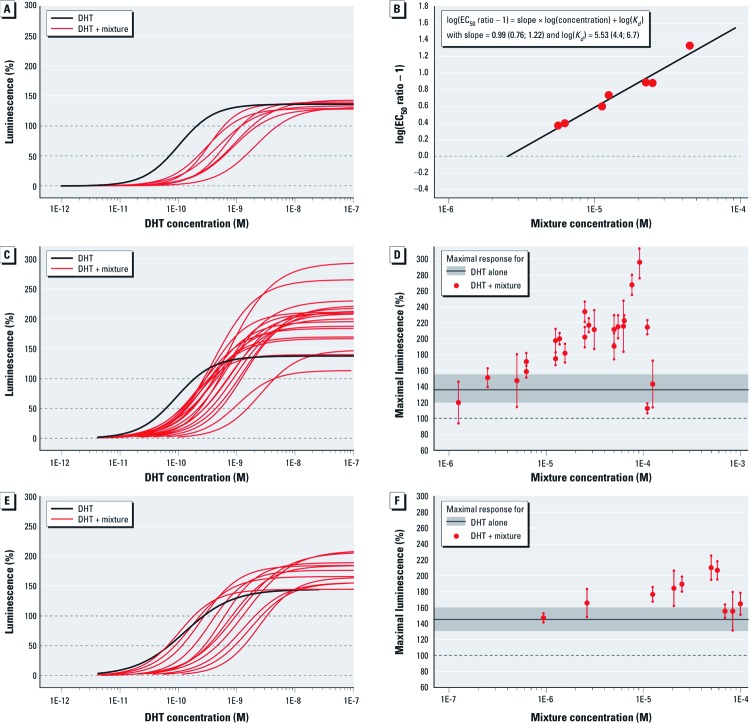
Antiandrogenic activity of DHT in the presence of fixed mixture concentrations of 8 pesticides (IC_10_ mixture ratio, *A*); 5 pesticides (IC_01_ mixture ratio, *C*); and 13 pesticides (IC_01_ mixture ratio, *E*). (*B*) Schild regression plot for the mixture of 8 pesticides. (*C,F*) Estimated maximal effect levels (mean and 95% CI) in response to the 5‑ and 13-component mixtures, respectively; gray shading indicates the average maximal effect level (and 95% empirical confidence belt) of DHT alone on the basis of all experiments.

We obtained similar results with a Schild plot analysis of the 8 mix, which was composed of “pure” AR antagonists. Increasing concentrations of the 8 mix shifted the DHT curve progressively towards lower concentrations, without affecting the maximal response of the agonist. The resulting Schild plot was linear, suggesting that the observed AR antagonistic effect of the mixture was indeed due to competitive AR antagonism without being confounded by multiple binding sites or pharmacokinetic interactions (Figure 2A,B).

However, in the presence of the 5 mix and 13 mix, the maximal effects observed at saturating DHT concentrations rose far above the levels normally seen with the agonist on its own (i.e., “supramaximal” effects) (Figure 2C,E). These supramaximal DHT responses increased with rising mixture concentrations until 100 µM (5 mix) and 70 µM (13 mix). Beyond these concentrations, a downturn of responses was observed (Figure 2D,F). This downturn corresponded with the cytotoxicity values obtained by analysis of the test mixture in the absence of DHT and thus can be explained in terms of this mechanism. Supramaximal effects violate one basic assumption of the Schild plot analysis, namely that an antagonist should not influence the maximal response of the agonist. Therefore, Schild plots could not be constructed for the 5 mix and 13 mix. These results show that the suppression of DHT effects seen with these two mixtures are not solely due to competitive receptor antagonism and suggest that more complex processes are operational at the receptor.

To investigate whether stimulation of the maximal response with the 5 mix and DHT was the result of cell proliferation, we used a *Renilla* luciferase construct in our assay. This construct produces luminescence in proportion to cell number, independent of AR activation. There was no dose–response relationship between rising concentrations of the 5 mix and *Renilla* luminescence of the MDA-kb2 cells and no differences in luminescence between ethanol only (mean ± SD, 3,335 ± 896) and the DHT background concentration only (3,036 ± 756). The same applied to DHT (3,059 ± 689), the positive control procymidone (4,115 ± 820), and to any concentration of the 5 mix with DHT (110 µM: 2,198 ± 418; 55.5 µM: 1,909 ± 399; 27.5 µM: 2,080 ± 359; 13.75 µM: 2,340 ± 379) and the 5 mix on its own (150–5.6 µM: 2,134 ± 322) (data not shown). This indicates that cell proliferation was not the cause of the increased luminescence observed with this mixture and DHT, and that the supramaximal responses were the consequence of phenomena at the receptor.

## Discussion

This is the first time that a mixture of widely used pesticides has been shown to produce combined AR antagonist effects that exceed the responses elicited by the most potent component alone. Furthermore, these mixture effects occurred in a quite predictable manner. The responses of the 8 mix composed of “pure” AR antagonists agreed very well with the combined effects predicted by CA. However, the combined effects seen with the two mixtures containing AR antagonists that also showed AR agonist properties (5 mix and 13 mix) were somewhat lower than anticipated by CA and fell between the “prediction window” boundaries defined by the predicted regression curves of CA and IA. These deviations are highly unlikely to be due to experimental artefacts because the mixtures were tested independently by different experimenters who prepared several independent mixture stock solutions.

By conducting Schild plot analysis, we were able to pinpoint competitive AR antagonism as a key factor that influenced agreement of the experimentally observed responses with the CA predictions. The combination of 8 “pure” AR antagonists (8 mix), well predicted by CA, produced Schild plots typical of competitive receptor antagonism. In contrast, anomalous supramaximal effects were observed with the 5 mix and 13 mix in experiments where increasing concentrations of these two mixtures were combined with DHT. These anomalies suggest that the 5 mix and 13 mix displaced DHT from the AR in ways not compatible with competitive antagonism. This allows us to infer that the lack of competitive AR antagonism is the likely cause of the observed deviations from CA. Chemicals that display mixed androgenic/antiandrogenic activity interact in ways with the amino acid residues in the AR binding domain that are distinct from those of “pure” AR antagonists (Tamura et al. 2006). Chemicals containing pyrimidine domains such as cyprodinil and pyrimethanil can cause AR antagonism via a non-ligand binding domain of the AR (Gunther et al. 2009). Pesticides of this kind formed a large proportion of the 5 mix and 13 mix, but were not present in the 8 mix. Although further mechanistic studies would be necessary to substantiate these ideas, we suggest that these modalities may play a role in the deviations from expected concentration additivity that we observed with the 5 mix and 13 mix. However, other explanations, such as stabilization of the AR-DNA binding complex or downstream effects of the signaling pathway may also be valid. In addition, there is some evidence that estrogenic supramaximal effects may be assay specific (Montaño et al. 2010), and although similar data are not available for AR antagonist assays, this is also a possible explanation for observed effects.

Deviations from expected additivity are interesting from a mechanistic viewpoint, but their relevance in relation to the application of CA or DA in risk assessment practice is not well defined. While it is reasonable that similarly acting pollutants should be assessed together, it is a matter for debate how chemicals should be grouped that do not match very narrowly defined criteria for similarity. Specifically, the question raised by our results is whether only “pure” AR antagonists that displace DHT in a competitive manner qualify for inclusion in groupings conforming to CA and whether, therefore, AR antagonists with AR agonistic properties should be excluded from joint assessments under CA principles. In resolving this issue, it is helpful to consider how combinations of AR antagonists behave *in vivo*. The applicability of CA for mixtures composed of AR antagonists was tested in a rat developmental toxicity model with flutamide, procymidone (both “pure” antagonists) and vinclozolin (which liberates metabolites that possess mixed AR antagonistic and agonistic properties) (Hass et al. 2007; Metzdorff et al. 2007). In these studies, the observed responses, including anogenital distance, reproductive organ weights, and prostate gene expression [PBP C3 (the prostate-specific binding protein polypeptide C3)] did not differ significantly from CA. For nipple retention, the observed response slightly exceeded the predicted mixture effects (Hass et al. 2007). Although it is not possible to arrive at firm conclusions based on these studies, it appears that the AR antagonist and agonist properties observed *in vitro* do have negligible impacts on the effects that are observed *in vivo*. A recent report has recommended that, despite the small deviations from CA that have sometimes been observed *in vitro* and *in vivo*, the evidence overwhelmingly suggests that it is a more accurate prediction model than IA (Kortenkamp et al. 2012). Furthermore, CA is a more conservative estimate of effect than IA, and thus CA would be protective for mixtures that fall in the “prediction window” (Kortenkamp et al. 2012). Therefore, we propose that CA is a suitable model for mixtures that contain AR antagonists with agonist properties, and that these chemicals should be grouped together with “pure” AR antagonists.

Early-life exposure is thought to be crucial for the development of abnormalities in male reproductive health (Skakkebaek et al. 2001). Fresh fruit and vegetables are consumed in high amounts by women and young children (Claeys et al. 2010), but these food items contain both the highest concentrations of single pesticides and the highest percentage with multiple residues (EFSA 2010; Inigo-Nunez et al. 2011, 87% of foodstuffs; Schiliro et al. 2011, 90% of foodstuffs). It is also interesting to note that a strong association between hypospadias and consumption of market fruit (odds ratio 5.10; CI: 1.31, 19.82) was reported for an agricultural population of Italy (Giordano et al. 2008). Fungicides were the most common pesticides detected in fruits and vegetables in several studies (Claeys et al. 2010; Inigo-Nunez et al. 2011; Schiliro et al. 2011), and they make up 9 of the 13 pesticides selected for testing in the present study. Although fungicides have broadly comparable use, as indicated by their global market share (22%) compared to herbicides (40%) and insecticides (29%) (Grube et al. 2011), fungicides are often applied post-harvest. As a result, their contribution to human exposures may well be higher than that of other pesticides. Furthermore, because of the rapidly evolving resistance of target organisms to fungicides, fungicides are recommended to be applied in mixtures for maximum effectiveness (Fungicide Resistance Action Committee 2010). For example, the commercial formulation “Switch” contains cyprodinil and fludioxonil (Syngenta 2011), “Forum” is composed of dithianon and dimethomorph (BASF 2010), “Justmeet” of fenhexamid and fludioxonil, and “Teldor Combi” contains fenhexamid and tebuconazole (Bayer 2011). There is a clear potential for human exposures via residues on foodstuffs, but to our knowledge, human biomonitoring data for the fungicides tested in this study are not available. There is also a lack of *in vivo* data for pesticides tested in this mixture (see Orton et al. 2011). Thus, it is currently not possible to ascertain the relationship between *in vitro* potency, *in vivo* effects, and exposure with adequate certainty, but such information is required if the risks to human health are to be properly assessed.

## Conclusions

Widely used pesticides act additively *in vitro* as AR antagonists. The less accurate predictability of mixtures containing antagonists that also have agonist activity may due to distinct action at the ligand-binding domain of the AR. Despite the unknown pharmacological cause of deviation from CA, it should still be used for risk assessment because of the minimal deviation observed and the protective (worst-case) nature of CA. It is well known that people are exposed to mixtures of pesticides, and therefore the additive nature of these pollutants is a cause for concern.

## Supplemental Material

(1.1 MB) PDF
