# Supplementary material for: Competitive Androgen Receptor Antagonism as a Factor Determining the Predictability of Cumulative Antiandrogenic Effects of Widely Used Pesticides
Source: Environ Health Perspect. 2012 Sep 10;120(11):1578–84. doi: 10.1289/ehp.1205391 (PMC3556629; doi:10.1289/ehp.1205391)
Supplement: (1.1 MB) PDF [file ehp.1205391.s001.pdf]

Supplemental Material

Competitive Androgen Receptor Antagonism as a Factor Determining the Predictability of Cumulative Antiandrogenic Effects of Widely Used Pesticides

Frances Orton, Erika Rosivatz, Martin Scholze and Andreas Kortenkamp

Contents

Table S1, components of test mixtures Page 2

Table S2, antiandrogenic activity, androgenicity and cytotoxicity of mixtures Page 3

Figure S1, flutamide Schild regression plot Page 4

Figure S2, cytotoxicity and androgenicity of mixtures Page 5

Supplemental Material, Table S1: Test mixtures

Component (by order of IC10)	Relative proportions (percentages)							
	Mixture of eight pesticides		Mixture of five pesticides		Mixture of 13 pesticides			
	IC01	IC10	IC01	IC10	IC01	IC10	IC20	IC50
Vinclozolin	-	-	0.12%	0.26%	0.08%	0.18%	0.21%	0.23%
Dimethomorph	1.22%	1.07%	-	-	0.40%	0.34%	0.34%	0.37%
Fludioxonil	3.13%	3.08%	-	-	1.02%	1.01%	1.04%	1.17%
Fenhexamid	1.67%	6.15%	-	-	0.54%	1.92%	2.53%	3.07%
Linuron	-	-	2.62%	2.81%	1.82%	1.92%	2.10%	2.78%
Tebuconazole	13.32%	11.88%	-	-	4.35%	3.77%	3.67%	3.55%
Imazalil	5.91%	11.91%	-	-	1.93%	3.76%	4.07%	3.61%
Phenylphenol	6.66%	12.69%	-	-	2.17%	3.98%	4.31%	4.14%
Pirimphos-methyl	21.79%	21.26%		-	7.11%	7.01%	7.23%	7.76%
Methiocarb	46.31%	31.96%	-	-	15.11%	9.93%	8.48%	6.34%
Chlorpropham	-	-	24.68%	16.79%	17.16%	11.46%	10.06%	8.75%
Cyprodinil	-	-	36.50%	33.08%	25.39%	22.59%	19.15%	12.26%
Pyrimethanil	-	-	36.08%	47.06%	25.10%	32.13%	36.82%	45.97%

Rounded values given for relative proportions

Supplemental Material, Table S2 – Receptor-mediated antiandrogenic activity, androgenicity and cytotoxicity of individual compounds and mixtures in the MDA-kb2

Substance (by order of IC10)		Concentration Response Function					Anti-androgenicity		Androgen	Cytotoxicity
		RM	$\hat{\theta}_1$	$\hat{\theta}_2$	$\hat{\theta}_{min}$	θ_{max}	IC10 (M), [CI]		EC10 (M)	EC10 (M)
Vinclozolin		logit	-18.48	-2.91	0.08	1	8.51E-8	[6.89E-8 - 1.03E-7]	1.70E-6	>5.0E-5
Dimethomorph		weibull	-10.09	-1.61	-0.04	1	1.59E-7	[1.28E-7 - 1.99E-7]	negative	> 2.5E-5
Fludioxonil		weibull	-7.41	-1.32	-0.29	1	4.61E-7	[3.43E-7 - 6.13E-7]	negative	1.83E-5
Fenhexamid		logit	-11.31	-2.28	-0.28	1	9.19E-7	[6.00E-7 - 1.36E-6]	negative	1.30E-5
Linuron		weibull	-6.91	-1.29	-0.13	1	9.53E-7	[6.21E-7 - 1.32E-6]	1.58E-6	>5.0E-5
Tebuconazole		weibull	-7.50	-1.47	-0.34	1	1.78E-6	[1.50E-6 - 2.06E-6]	negative	3.13E-5
Imazalil		logit	-14.96	-3.03	-0.28	1	1.78E-6	[1.44E-6 - 2.16E-6]	negative	1.58E-5
Phenylphenol		logit	-15.99	-3.17	0.03	1	1.90E-6	[1.21E-6 - 2.52E-6]	negative	> 5.0E-5
Pirimphos-methyl		weibull	-6.04	-1.28	-0.42	1	3.18E-6	[2.26E-6 - 4.25E-6]	negative	>5.0E-5
Methiocarb		weibull	-10.22	-2.09	-0.23	1	4.78E-6	[3.30E-6 - 5.45E-6]	negative	>4.6E-5
Chlorpropham		weibull	-10.83	-2.21	0.05	1	5.22E-6	[4.71E-6 - 6.04E-6]	1.05E-6	>5.0E-5
Cyprodinil		logit	-24.90	-5.43	0.08	1	1.07E-5	[8.69E-6 - 1.74E-5]	1.00E-6	>5.0E-5
Pyrimethanil		weibull	-3.67	-0.98	-0.76	1	1.41E-5	[9.12E-6 - 2.18E-5]	1.68E-5	>1.25E-4
Mixtures with ratio as defined in Tab.S1										
8Mix :	IC01	weibull	-5.14	-1.07	-0.75	1	1.65E-6	[1.11E-6 - 2.21E-6]	negative	7.74E-5
	IC10	weibull	-6.23	-1.23	-0.38	1	1.49E-6	[1.23E-6 - 1.85E-6]	negative	6.08E-5
5Mix:	IC01	weibull	-8.69	-1.93	-0.29	1	1.03E-5	[8.25E-6 - 1.21E-5]	7.35E-6	7.01E-5
	IC10	weibull	-6.95	-1.57	-0.41	1	8.97E-6	[6.77E-6 - 1.20E-5]	1.32E-5	7.41E-5
13Mix:	IC01	weibull	-6.88	-1.49	-0.28	1	5.56E-6	[4.38E-6 - 7.39E-6]	1.78E-6	7.50E-5
	IC10	weibull	-4.48	-1.05	-0.95	1	5.20E-6	[3.47E-6 - 7.28E-6]	2.90E-6	6.25E-5
	IC20	logit	-10.81	-2.40	-0.15	1	3.25E-6	[2.28E-6 - 4.91E-6]	6.25E-6	7.50E-5
	IC50	logit	-11.77	-2.68	-0.16	1	5.41E-6	[3.70E-6 - 7.44E-6]	1.56E-5	8.13E-5

IC10: concentration that inhibits the androgenicity of 0.25 nM DHT by 10%; EC10: concentration that produces a 10% effect. Values in brackets denote the upper and lower limits of the approximate 95% confidence interval; the column "RM" indicates the mathematical regression function as defined at Scholze *et al.* (2001): $\hat{\theta}_1, \hat{\theta}_2, \hat{\theta}_{min}$ estimated model parameters, given for concentrations expressed in M (rounded values), θ_{max} were not estimated, but set to 1 relating to the mean value of the DHT controls (individual data first reported in Orton et al. 2011)

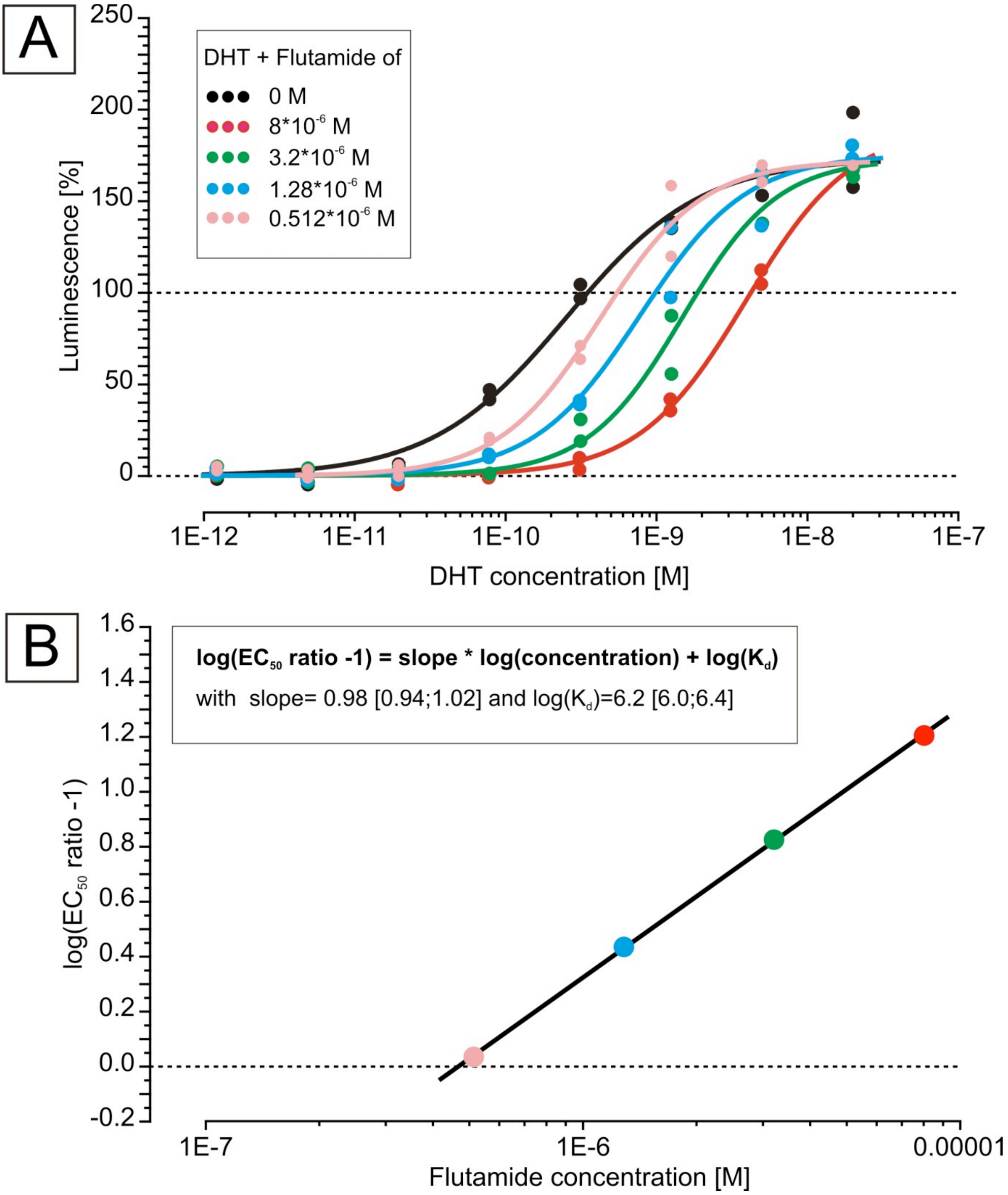

Supplemental Material, Figure S1*: Concentration-response curves for DHT in the presence of fixed flutamide concentrations (A) and corresponding Schild regression plot (B).*

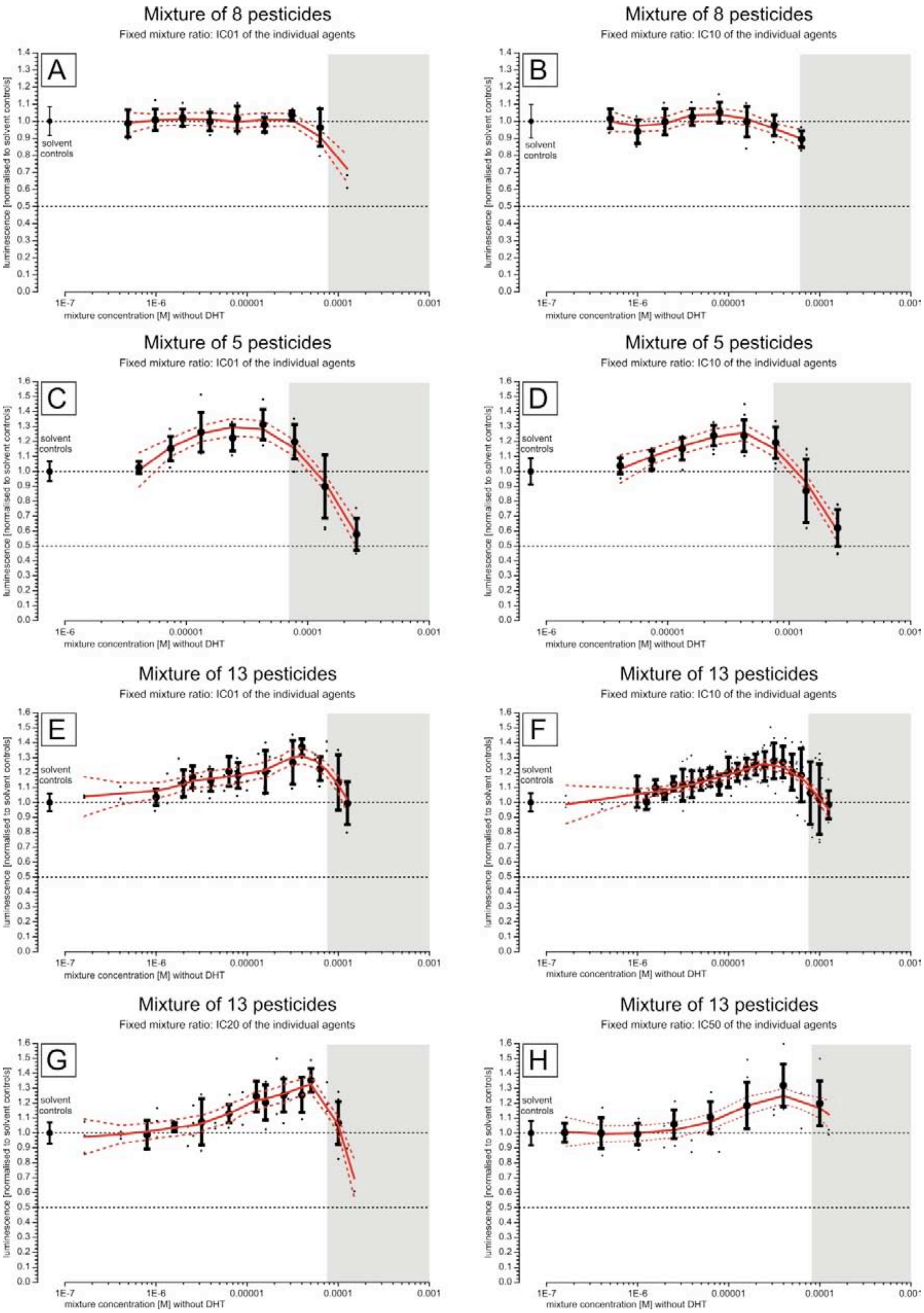

***Supplemental Material, Figure S2**: Tested mixtures without DHT - Observed luminescence of mixtures with 8 pesticides composed in the ratio of their individual IC01 values (A) and IC10 values (B), with 5 pesticides mixed in the ratio of their individual IC01 values (C) and IC10 values (D), and with 13 pesticides mixed in the ratio of their individual IC01 values (E), IC10 values (F), IC20 values (G) and IC50 values (H). Observed effects are from at least three independent mixture experiments and shown as mean ± standard deviation, non-parametric regression fit of the observed effects is shown as red line, with the dotted red line indicating the corresponding 95% confidence belt. Cytotoxicity is highlighted as grey area (see Material & Methods for more details).*
